# The Impact of Coronavirus Disease 2019 Lockdown on Athletes’ Subjective Vitality: The Protective Role of Resilience and Autonomous Goal Motives

**DOI:** 10.3389/fpsyg.2020.612825

**Published:** 2021-02-10

**Authors:** Natalia Martínez-González, Francisco L. Atienza, Inés Tomás, Joan L. Duda, Isabel Balaguer

**Affiliations:** ^1^Department of Social Psychology, Faculty of Psychology, University of Valencia, Valencia, Spain; ^2^Department of Personality, Evaluation and Psychological Treatment, Faculty of Psychology, University of Valencia, Valencia, Spain; ^3^Department of Methodology of the Behavioral Sciences, Faculty of Psychology, University of Valencia, Valencia, Spain; ^4^School of Sport, Exercise and Rehabilitation Sciences, University of Birmingham, Birmingham, United Kingdom

**Keywords:** resilience, goals, vitality, well-being, athletes, motivation, COVID-19, lockdown

## Abstract

The lockdown resulting from coronavirus disease 2019 (COVID-19) has had a huge impact on peoples’ health. In sport specifically, athletes have had to deal with frustration of their objectives and changes in their usual training routines. The challenging and disruptive situation could hold implications for their well-being. This study examined the effect of the COVID-19 lockdown on changes in athletes’ reported eudaimonic well-being (subjective vitality) and goal motives (autonomous and controlled) over time (i.e., pre-lockdown and during lockdown). The relationship of resilience to changes in subjective vitality was also determined, and changes in athletes’ goal motives were examined as potential mediators. Participants were 127 Spanish university athletes aged between 18 and 34 years (*M* = 21.14; *SD* = 2.77). Approximately 4 months before the start of the lockdown in Spain (T1), athletes responded to a questionnaire assessing their resilience, goal motives, and subjective vitality. Around 6 months later into the lockdown period (T2), athletes’ goal motives and subjective vitality were assessed again. Growth modeling using hierarchical linear models revealed a significant decrease of autonomous goal motives and subjective vitality during the lockdown, but athletes did not show change over time in controlled goal motives. Path analysis, adjusting T2 measures for their corresponding T1 measures, showed that resilience significantly predicted changes in athletes’ autonomous goal motives, which then accounted for changes in subjective vitality. The indirect effect was significant. Resilience did not predict changes in athletes’ controlled goal motives. However, changes in controlled goal motives negatively predicted changes in subjective vitality during lockdown. The findings suggest negative impacts of the COVID-19 lockdown on athletes’ goal motives and eudaimonic well-being. Results also support the hypothesized mediational role of autonomous goal motives in the relationship between resilience and subjective vitality during the lockdown. As such, findings confirm the relevance of resilience to a key feature of athletes’ eudaimonic well-being and the importance of enhancing their autonomous goal striving.

## Introduction

The coronavirus disease 2019 (COVID-19) has become a health global issue, with millions of confirmed cases in many countries around the world (Satici et al., 2020). The pandemic has put at risk people’s physical health and functioning, and also their psychological health and well-being (Bakioğlu et al., 2020). Research has reported a wide range of associated negative psychological responses such as anxiety and depression (Harper et al., 2020; Taylor et al., 2020), as well as a decrease in emotional well-being (Yang and Ma, 2020). The risk of being infected by COVID-19 and/or passing the virus onto others, together with the sense of social isolation caused by the lockdown and quarantine, can escalate people’s anxiety and fear (Rubin and Wessely, 2020).

One of the populations affected by the pandemic has been athletes, who also have experienced cancelation of important competitive events, restrictions to training, and disruption of everyday life. During the lockdown period, the measures and restrictions varied from country to country. In some countries like Spain, sport and physical activities were canceled because these were not considered as essential activities. For athletes, their only alternative to train therefore was to train at home, with the limitations that this implies. In these circumstances, athletes have encountered issues not only related to their options to compete and maintain their usual training routines but also challenges presented by social isolation and need to distance from partners and teammates (Schinke et al., 2020). This has resulted in a decrease in athletes’ training intensity and training volume and other consequences such as a reduction of sleep quality (Mon-López et al., 2020) and changes in their dietary patterns (Roberts et al., 2020). Athletes have also reported COVID-19-related psychological distress and worries about one’s sport and about one’s own future in sport (Håkansson et al., 2020). Recent evidence suggests that the challenges brought about by COVID-19 have physical, nutritional, and psychological consequences that may affect their overall health (Pillay et al., 2020).

Understanding the psychological mechanisms that allow athletes to maintain eudaimonic well-being in a stressful situation can contribute to greater insight into contributors to their mental health. Such information is relevant to the development and delivery of preventive, health-based psychological interventions in the sport setting.

Eudaimonic well-being is referred to actualizing one’s human potentials and is considered a positive subjective state that is the product of the pursuit of self-realization rather than the objective being sought (Waterman, 2007). As Waterman pointed out, eudaimonia is stronger when it is associated with opportunities to develop one’s best potentials, with investing a great deal of effort, with having clear goals, and with feeling challenged. It can be considered that lockdown period has restricted, for several months, these opportunities. A key construct that has traditionally been used as an indicator of people’s positive mental health and eudaimonic well-being specifically is subjective vitality (Ryan and Deci, 2001). Ryan and Frederick (1997) defined subjective vitality as “the experience of having positive energy available to or within the regulatory control of one’s self” (Ryan and Frederick, 1997, p. 530). This concept not only involves somatic factors, but psychological factors are also strongly implicated. When individuals indicate that they have high subjective vitality, they feel alive, vigorous, and energetic. In fact, subjective vitality has been considered as “perhaps the most general characteristic of a fully functioning person” (Ryan and Deci, 2017, p. 256). In sport (e.g., Mack et al., 2011; Adie et al., 2012; Castillo et al., 2017), it has been found that subjective vitality is positively related to other indicators of well-being (e.g., life satisfaction and global self-esteem) and negatively related to indicators of ill-being (e.g., emotional and physical exhaustion). One aim of the present study is to examine whether there was a change in athletes’ reported subjective vitality from before to during the lockdown.

Ryan and Frederick (1997) pointed out that when people are involved in activities driven by volition, these activities catalyze energy, whereas if the same activities are driven by external motives, such feelings of energy can be depleted. Autonomous self-regulation implies less control and inhibition than when people are involved in activities fueled by more external or internally contingent forces. Drawing from self-determination theory (SDT; Deci and Ryan, 1985, 2000), it is expected that when autonomous, rather than controlled motivation, directs human behavior, reported vitality is higher. Consonant with SDT tenets, past research has revealed athletes’ subjective vitality to be positively predicted by autonomous motivation (e.g., Mouratidis et al., 2008; Gunnell et al., 2014).

Athletes, as is the case for humans in general, are constantly pursuing goals that specify and direct their behavior (Sheldon, 2014), although they sometimes are not explicitly aware of those goals (Emmons, 1989). From this perspective, it could be assumed that even during the lockdown period, athletes directed their behavior (consciously or unconsciously) to achieve some goals. Grounded within the larger SDT framework, the self-concordance model (SCM; Sheldon and Elliot, 1999) focuses on the motives underlying personal goal striving and allows, through an idiographic methodology proposed by Sheldon (2002), the examination of specific goals generated by the person and their underlying motivational regulations. According to the SCM, goals can be guided by two different forms of motivation termed as autonomous or controlled, depending on whether goal motives are more or less concordant with the person. Autonomous goal motives are based on personal interest, enjoyment, or perceived importance, whereas controlled goal motives are regulated by external or internal pressures and contingencies that are related with social approval (Sheldon and Elliot, 1999). Therefore, autonomous goal motives are intrinsically motivated or integrated with a person’s sense of self, whereas controlled goal motives are based on external and introjected regulations (Martela et al., 2016).

Empirical research has supported the adaptive role of autonomous goal motives in comparison with controlled goal motives across different contexts (Healy et al., 2020). For example, Judge et al. (2005) found that more self-concordant goals in the work domain corresponded with greater job and life satisfaction. In sport, the SCM was successfully adapted and tested by Smith et al. (2007). Smith et al. found that autonomous goal motives positively predicted psychological well-being (operationalized in respect to more hedonic aspects of well-being and ill-being), whereas controlled goal motives emerged as a negative predictor. Autonomous goal motives have been positively related with a range of self-regulatory processes and with adaptive coping strategies (Sanjuán and Ávila, 2018), as well as to greater persistence in goal pursuit (Ntoumanis et al., 2014), goal-directed effort and goal attainment (Smith et al., 2011), and well-being (Smith et al., 2011; Healy et al., 2014). Conversely, controlled goal motives have been unrelated to goal attainment (Ntoumanis et al., 2014) and positively related to reported ill-being (Healy et al., 2014; Gaudreau and Braaten, 2016). In the present study, the form of motivation (autonomous and controlled) underlying athletes’ goals during the lockdown was examined in relation to their feelings of subjective vitality.

Previous evidence suggests that, aligned with the principles of SDT, controlled and autonomous goal motives can be influenced by features of the social environment as well as by personal dispositions (Healy et al., 2018). As for the social environment, the lockdown and its consequences in athletes’ daily activities could affect the self-concordance of their goals, diminishing autonomous goal motives and increasing the controlled ones. Literature has largely demonstrated that when social contexts frustrate the humans’ basic psychological needs of autonomy, competence, and relatedness, the autonomous motivation tends to decrease, whereas the controlled motivation tends to flourish (Deci and Ryan, 2000; Ryan and Deci, 2017). During the lockdown, athletes have had less opportunities for choice in general, and their relationships have been affected because of their isolation. To this must be added that in this situation the external pressures on human agency increased significantly, especially those exercised by the government and authorities, putting at risk a more autonomous functioning. Regarding the personal dispositions, recent studies have analyzed the mediating role of motivation in the relationship between personality dispositions and indicators of well-being and/or ill-being, including constructs such as perfectionism (Atienza et al., 2020) and resilience (León-Guereño et al., 2020).

The present study focuses on the role of resilience, not only as a personality disposition that should differentially correspond to goal motives, but also as a likely positive predictor of athletes’ vitality. Considering that the characteristics and correlates of this personality disposition would be expected to further understand why, in adverse situations, the subjective vitality of athletes is more or less affected.

Resilience is defined as an individual’s ability to cope effectively and overcome life’s adversities (Rutter, 1993). This personal disposition allows people to be more likely to achieve stable healthy functioning and cope more optimally with stress (Devi, 2020), in part due to its role as a buffer to disease and links to an adaptive immunological system. Characteristics such as sense of control, optimism, and persistence among others are considered typical attributes of resilient people (Grant and Kinman, 2014; Collins, 2015). Previous research has shown resilience to positively correlate with adaptive behaviors within adverse situations (Olsson et al., 2003). In addition, past studies suggest that individuals with high levels of resilience have more positive cognitions and higher levels of life satisfaction (Mak et al., 2011), self-esteem (Benetti and Kambouropoulos, 2006), and perceived self-efficacy (Schwarzer and Warner, 2013). Within work based on SDT, research has focused on studying contextual need support as a positive predictor of resilience (e.g., Vansteenkiste and Ryan, 2013). Little is known about whether and how resilience corresponds to heightened well-being in athletes, particularly during uncertain, challenging times.

In sport, competition stimulates athletes to use psychological abilities and efforts to use effective coping strategies to improve their performance in often stressful conditions (Nicholls and Polman, 2007). Resilience is one of these abilities and can help athletes adapt to difficult situations and promote their personal growth (Galli and Vealey, 2008). This ability to “bounce-back” from a defeat or disappointment in order to overcome future challenges contributes to the psychological well-being and performance of athletes (Gupta and Sudhesh, 2019). In this line, past sport research suggests that resilience is positively related to other psychological resources such as self-efficacy (Cardoso and Sacomori, 2014), optimism and coping strategies (Belem et al., 2014), and intrinsic motivation and self-regulation (Brown et al., 2015). In contrast, resilience in athletes has been negatively related to pessimism, self-blame, anxiety, and depression (Sarkar and Fletcher, 2014) as well as to stress and burnout (Codonhato et al., 2018; Wagstaff et al., 2018). In the same way, previous literature in university student athletes demonstrated that resilience had a positive relationship with performance and psychological well-being and a negative relationship with psychological disorders (Hosseini and Besharat, 2010). More recently, González et al. (2019) found resilience to positively predict enjoyment and negatively predict boredom in sport.

Due to the importance of subjective vitality for athletes in their daily and sporting lives and particularly in periods of challenge and/or crisis (such as lockdown by COVID-19), it is important to understand the mechanisms by which personal dispositions (i.e., in this case, resilience) may be predictive of changes in athletes’ reported vitality over time. In this research, athletes’ goal motives (autonomous and controlled) as possible mechanisms by which resilience can predict changes in subjective vitality during the COVID-19 pandemic was examined. A number of recent studies, conducted across different populations and countries, have described the impact of COVID-19 lockdown on athletes (e.g., Håkansson et al., 2020; Mon-López et al., 2020; Pillay et al., 2020; Roberts et al., 2020). However, to our knowledge, there has been no research about the role of resilience and goal motives on changes in athlete’s subjective vitality during lockdown.

Specifically, the two main objectives of this study were as follows:

Objective 1: Examine the effect of the COVID-19 lockdown on changes in athletes’ reported eudaimonic well-being (subjective vitality) and goal motives (autonomous and controlled), by comparing their scores before (T1) and during the lockdown (T2).

Objective 2: Study the predictive role of resilience (T1) to changes in subjective vitality (T2 controlling for T1) and to test whether athletes’ goal motives serve as mediators of this relationship (T2 controlling for T1).

Based on previous evidence and theoretical tenets, two hypotheses were formulated:

Hypothesis 1: There will be significant differences between T1 and T2 athletes’ scores in subjective vitality and goal motives. Specifically, it is expected that subjective vitality and autonomous goal motives will decrease during the lockdown. Conversely, it is hypothesized that controlled goal motives will increase during these adverse circumstances.

Hypothesis 2: Athletes’ resilience (T1) will predict changes in subjective vitality (T2 controlling for T1) during the lockdown, through changes in goal motives (T2 controlling for T1). Specifically, it is expected that resilience will positively predict changes in autonomous goal motives, which in turn will positively predict changes in subjective vitality. Conversely, resilience will negatively predict changes in controlled goal motives, which in turn will negatively predict changes in subjective vitality.

## Materials and Methods

### Participants

An *a priori* power analysis using GPower (Faul et al., 2009) indicated that 125 participants were necessary to attain a statistical power level of 0.80 in regard to detecting the expected relationships. One hundred twenty-seven athletes from two different Valencian universities (Spain) were recruited to participate in the study [age range of 18–34 years (*M* = 21.14, *SD* = 2.77)]. Females constituted 50% of the sample. All the athletes competed in the Autonomic Championship of University Sports (CADU) and participated in one of a variety of team sports (e.g., basketball, handball, football, rugby, and volleyball).

### Procedure

After obtaining ethical approval from the Human Research Ethics Committee of the university, researchers contacted the directors of each sport service to present the project and request their willingness to allow their athletes to participate. Taking into account the characteristics of the Spanish language, which has different verb conjugations to make reference to male or female, the questionnaires were adapted for each sex.

Before the data collection, participants received brief instructions and signed an informed consent to voluntarily participate in the research. Confidentiality and anonymity were guaranteed through an identification code, which permitted monitoring of the responses of the participants over time. The first assessment was carried out at the beginning of the university season in November, and the second assessment took place in May, during the period of lockdown resulting from COVID-19. At T1, data were collected from the athletes in small groups at the sport facilities. At T2, due to the lockdown, questionnaires were adapted to be completed through an online survey. All the athletes completed the package of questionnaires measuring the variables of interest at both T1 and T2 (before and during the lockdown).

### Instruments

The Spanish version (Castillo et al., 2017) of the Subjective Vitality Scale (SVS; Ryan and Frederick, 1997) was used to measure the athletes’ subjective vitality. The SVS comprised six items (e.g., “I feel alive and vital”) to evaluate the subjective experience of being full of energy and alive. All responses were provided on a 7-point Likert scale from 1 (*not at all true*) to 7 (*very true*). The reliability and validity of this scale have been supported in previous studies conducted in the sport domain and in the case of Spanish athletes, with alphas ranging between 0.77 and 0.89 (e.g., Álvarez et al., 2012; Balaguer et al., 2012; González et al., 2015; Balaguer et al., 2018).

Resilience was assessed via the Spanish version (González et al., 2019) of the Resilience Scale developed by Wagnild and Young (1993). The scale has 25 items grouped into two subscales: personal competence (17 items; e.g., “I usually manage one way or other”) and acceptance of oneself and life (8 items; e.g., “I get along with myself”). Participants responded on a Likert scale with a range from 1 (*strongly disagree*) to 7 (*strongly agree*). Following previous literature, a global resilience score was computed adding responses provided to both subscales (Wagnild and Young, 1993; Ruíz et al., 2012). Recently, González et al. (2019) used the scale in the sport domain, obtaining evidence of adequate validity and reliability (α = 0.86).

Personal goal motives items were adapted for use with Spanish athletes drawing from the ideographic methodology employed in previous self-concordance research (Sheldon, 2002). Most of past studies assessed more than one self-generated goal by participant, so the reliability of personal goal motives was obtained through the intraclass correlation coefficients (ICCs). This methodology has been marked by adequate validity and reliability (ICC = 0.87–0.95) and has been used in previous sport studies conducted in other countries (e.g., Smith et al., 2011; Smith and Ntoumanis, 2014). Based on this methodology, but adapting it for measure only one goal, in the present study, athletes were asked to identify their most important personal goal in relation with sport that they were currently pursuing. To assess the motives underlying goal striving, athletes rated the extent that they were striving for their current goal with extrinsic (two items; e.g., “Because someone else wants you to”), introjected (two items; e.g., “Because you would feel ashamed, guilty, or anxious if you didn’t”), identified (two items; e.g., “Because you personally believe it’s an important goal to have”), and intrinsic (two items; e.g., “Because of the fun and enjoyment the goal provides you”) motives. Responses were given on 7-point Likert scale ranging from 1 (*not at all*) to 7 (*very much so*). Autonomous motives were computed from intrinsic and identified motives, and controlled motives were calculated from introjected and extrinsic motives, consistent with past SCM research (e.g., Healy et al., 2014).

### Data Analysis

The IBM SPSS Statistics 25 software package was used to conduct preliminary analyses, i.e., calculate descriptive statistics, internal consistency of the scales (Cronbach’s alpha), and Pearson correlation analyses. The change over time (before and during the lockdown) on reported goal motives (autonomous and controlled) and subjective vitality (Hypothesis 1) was tested through growth modeling (Duncan et al., 2013) using hierarchical lineal models (Bliese and Ployhart, 2002; Heck et al., 2013). The following models for each of the three aforementioned variables were tested: Model 1 (M1) or “random intercept model” to estimate within-individual variance (σ^2^) and between-individual variance (σ^2^_τ00_), as well as the ICCs (Bliese, 2000); Model 2 (M2) or “linear growth model,” to check linear change over time (γ_10_); and Model 3 (M3) or “random slope model,” to test whether the linear trajectories varied across individuals (σ^2^_τ11_).

To test Hypothesis 2, path analysis was conducted using Mplus (Version 7; Muthén and Muthén, 2012). To determine the fit of the model, different fit indices were examined, such as chi-square (χ^2^), the comparative fit index (CFI), the Tucker–Lewis index (TLI), the standardized root mean square residual (SRMR) index, and the root mean square error of approximation (RMSEA) index. A non-significant χ^2^-value (*p* > 0.05) indicated that the path model fit the data well (Shah, 2012). Values of CFI and TLI of 0.95 or higher are considered acceptable (Hu and Bentler, 1999). For SRMR and RMSEA, values of 0.05 or lower indicate good model fit. Within the analyses, athletes’ reported goal motives and subjective vitality were controlled for their measures in T1 (i.e., T2 measures were adjusted in regard to their corresponding T1 measures), so that the effective outcome measures can be considered measures of change in the variable in question (Finkel, 1995). This methodology to measure the change in targeted variables has been widely applied in previous studies (e.g., Papaioannou et al., 2004; Chen et al., 2011; Balaguer et al., 2012).

## Results

### Preliminary Analysis

Prior to the main analysis, missing data and outliers were analyzed. The criterion applied for accept missing data was that omitted values were below 5% in each variable (e.g., Graham and Hofer, 2000). In this study, this criterion was met, being that the observed percentage of missing data was too small (0.5%) to be a potential problem. Outliers were analyzed in the measurements taken in both T1 and T2 using Z-scores, and the criterion applied was that values higher than ±3.29 were considered extreme (Tabachnick and Fidell, 2007, 2013; Field, 2013). With the use of the univariate trimming method, analyses identified and removed four participants from the sample, because they showed extreme values in autonomous goal motives (three participants) and resilience (one participant).

### Descriptive Statistics, Reliabilities, and Bivariate Correlations

Results of the descriptive analyses, scale reliability coefficients, and bivariate correlations are presented in Table 1. Skewness and kurtosis coefficients were in the recommended range of (−1, 1) for normal distributions (Muthén and Kaplan, 1985, 1992; Ferrando and Anguiano-Carrasco, 2010). In addition, Cronbach’s alpha coefficients showed satisfactory reliability for all the scales, except for autonomous goal motives, which were below the 0.7 criterion (0.62). Mean scores indicated that, overall, athletes had high levels of resilience. Moreover, athletes as a group were marked by high levels of subjective vitality in T1, but in T2, the scores were low. At T1 and T2, athletes scored high in autonomous goal motives and low in controlled goal motives.

**TABLE 1:** Descriptive statistics, reliabilities, and bivariate correlations for variables at T1 and T2.

	Range	*M*	*SD*	α	Skewness	Kurtosis	1	2	3	4	5	6	7
Resilience (T1)	1–7	5.56	0.63	0.89	−0.29	−0.25	–						
Autonomous goal motives (T1)	1–7	6.04	0.77	0.62	−0.87	0.37	0.34**	–					
Autonomous goal motives (T2)	1–7	5.74	1.04	0.71	−0.90	0.66	0.30**	0.08	–				
Controlled goal motives (T1)	1–7	2.53	1.23	0.73	0.69	−0.17	−0.25**	−0.17	0.02	–			
Controlled goal motives (T2)	1–7	2.73	1.46	0.77	0.65	−0.18	−0.20*	0.01	−0.14	0.24**	–		
Subjective vitality (T1)	1–7	5.01	1.06	0.87	−0.32	−0.33	0.43**	0.12	0.18*	−0.11	−0.15	–	
Subjective vitality (T2)	1–7	3.69	1.26	0.89	0.08	−0.57	0.22*	−0.05	0.26**	−0.14	−0.31**	0.27**	–

*p < 0.05; **p < 0.01.

Bivariate correlations showed that resilience measured in T1 was significantly and positively related with subjective vitality at T1 and T2. Moreover, resilience was positively related with autonomous goal motives at T1 and T2, whereas its relationship with controlled goal motives was negative at the two time points. Regarding goal motives, autonomous goal motives at T2 correlated positively with subjective vitality, whereas controlled goal motives correlated negatively with this well-being indicator, although these relationships were not significant at T1. In addition, consistent with previous research, autonomous goal motives and controlled goal motives were unrelated at T1 and T2 (Koestner et al., 2008; Smith and Ntoumanis, 2014). Finally, when comparing over time, autonomous goal motives at T1 and T2 did not show significant correlations, whereas controlled goal motives at T1 and T2 correlated positively. Reported subjective vitality was also significantly and positively correlated with levels at T1 and T2.

### Differences Before and During Lockdown

Results from M1 showed that the within-individual variance over time was statistically significant for autonomous goal motives (σ^2^ = 0.81, *p* < 0.01), controlled goal motives (σ^2^ = 1.40, *p* < 0.01), and subjective vitality (σ^2^ = 1.80, *p* < 0.01). Additionally, between-individual variance was also statistically significant for controlled goal motives (σ^2^_τ00_ = 0.42, *p* < 0.05), and subjective vitality (σ^2^_τ00_ = 0.36, *p* < 0.01), indicating that there were cross-time differences in athletes’ means on both variables. Between-individual variance was not statistically significant for autonomous goal motives (σ^2^_τ00_ = 0.04, *p* > 0.05). Furthermore, the ICC values indicated that the percentage of total variance produced by differences across individuals (between-individual variation) were as follows: autonomous goal motives = 4.8%; controlled goal motives = 23%; and subjective vitality = 17%.

Results from M2 indicated a significant decrease in autonomous goal motives (γ_10_ = −0.30, *p* < 0.01) and subjective vitality (γ_10_ = −1.33, *p* < 0.01) during the lockdown; however, there was no significant change over time in controlled goal motives (γ_10_ = 0.20, *p* > 0.05). Additionally, results from M3 revealed that there were no differences in the linear growth trajectories across individuals for controlled goal motives (σ^2^_τ11_ = 0.61, *p* > 0.05), and subjective vitality (σ^2^_τ11_ = 0.46, *p* > 0.05). There was significant variability, however, in the rate of change for autonomous goal motives (σ^2^_τ11_ = 0.48, *p* > 0.01). See Table 2 for the results of M1, M2, and M3.

**TABLE 2:** Change in goal motives (autonomous and controlled), and subjective vitality over time.

	Autonomous goal motives		Controlled goal motives		Subjective vitality	
			
Parameter	Estimate	SE	Estimate	SE	Estimate	SE
**M1. Random intercept model**						
Within-individual variance (σ^2^)	0.81**	0.10	1.40**	0.18	1.80**	0.16
Between-individual variance (σ^2^_τ00_)	0.04	0.08	0.42*	0.17	0.36**	0.13
**M2. Linear growth model**						
Intercept (γ_00_)	6.04**	0.08	2.53**	0.12	5.01**	0.10
Time (γ_10_)	−0.30**	0.11	0.20	0.15	−1.33**	0.13
**M3. Random slope model**						
Intercept (γ_00_)	6.04**	0.07	2.53**	0.11	5.01**	0.10
Time (γ_10_)	−0.30**	0.11	0.20	0.15	−1.33**	0.13
Slope_(σ^2^τ11)_	0.48**	0.16	0.61	0.32	0.46	0.24

SE, standard error. *p < 0.05; **p < 0.01.

### Path Analysis

**FIGURE 1:**
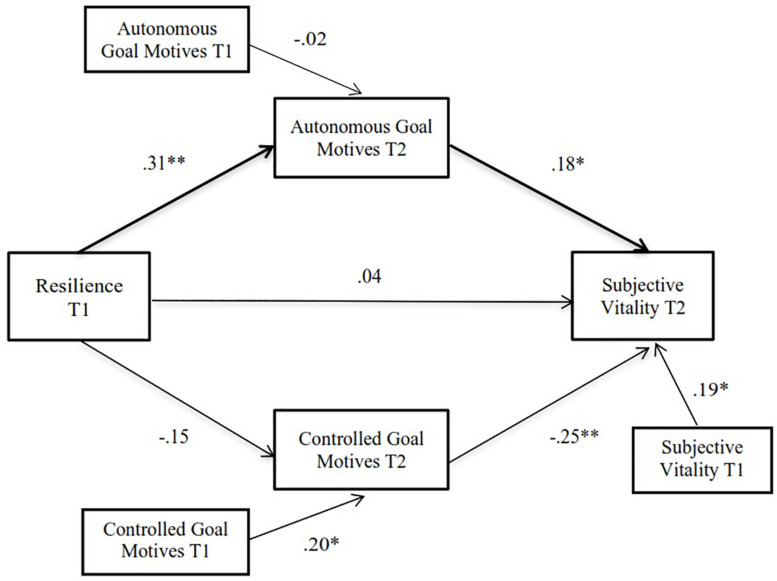
Path model of the associations between resilience, autonomous and controlled goal motives and subjective vitality over time. Statistics are standardized regression coefficients. Bold lines represent significant indirect paths. ^∗^*p* < 0.05; ^∗∗^*p* < 0.01.

The hypothesized model (see Figure 1) presented an adequate fit to the data: χ^2^(7) = 7.33, *p* > 0.05, RMSEA = 0.020, CFI = 0.991, TLI = 0.981, and SRMR = 0.048. The results, controlling for T1 variable values, showed that resilience did not directly predict changes in subjective vitality (β = 0.04, *p* > 0.05). However, resilience in T1 positively predicted changes in athletes’ autonomous goal motives (β = 0.31, *p* < 0.01), which in turn positively predicted changes in subjective vitality during lockdown (β = 0.18, *p* < 0.05). These results indicate that athletes with lower resilience reported a more pronounced decrease in autonomous goal motives over time and that athletes with greater decreases in autonomous goal motives over time exhibited more marked decreases in subjective vitality.

Results also revealed that resilience in T1 did not predict changes in athletes’ controlled goal motives (β = −0.15, *p* > 0.05). However, changes in athletes’ controlled motives negatively predicted changes in subjective vitality (β = −0.25, *p* < 0.01), indicating that athletes with greater decreases in controlled goal motives over time also reported less decreases in subjective vitality.

In order to test the hypothesized mediational effects, the significance of indirect effects was tested by bias-corrected (BC) bootstrap 95% confidence intervals (CIs) and its effect size with the P_M_ value or mediation ratio, which has been proposed as one of the effect size measures for mediation models (Preacher and Kelly, 2011). The indirect effect of resilience on subjective vitality through autonomous goal motives was significant (IE_a1b1_ = 0.11; BC bootstrap 95% CI = [0.02, 0.30]; P_M_ = 0.58), with the P_M_ value indicating that around 58% of the total effect of resilience on subjective vitality was mediated through autonomous goal motives. However, the expected mediational role of controlled goal motives was not supported (IE_a2b2_ = 0.07; BC bootstrap 95% CI = [−0.002, 0.25]; P_M_ = 0.47).

## Discussion

During the uncertainty and lockdown restrictions caused by the COVID-19 pandemic, athletes had to dramatically adapt their daily routines and functioning in regard to their sport training, planned competitions, and their lives in general (Pillay et al., 2020; Schinke et al., 2020). Given these challenging circumstances, the main purpose of this study was to analyze through a time-lagged design the impact of lockdown on an indicator of athletes’ eudaimonic well-being (i.e., subjective vitality) and goal motives. There was also interest in examining the role of dispositional resilience and the form of motivation (i.e., autonomous, controlled) underlying athletes’ goals in relation to athletes’ changes in their subjective vitality during this new and uncertain situation. Specifically, potential changes in athletes’ goal motives and their reported subjective vitality were examined, comparing their scores before and during the lockdown (Objective 1). Moreover, this study determined whether resilience predicted changes in subjective vitality, and this study tested the potential mediating role of changes in athletes’ goal motives in regard to this relationship (Objective 2).

With respect to the first hypothesis, results revealed a significant decrease in both athletes’ subjective vitality and autonomous goal motives during the lockdown (when contrasted with pre-lockdown values), whereas no significant change was found in controlled goal motives. In Spain, during the lockdown, athletes were not able to leave their house. They lived in a situation where they were socially restricted in their usual day-to-day activities as athletes and of course, as people. They had to organize their training and their everyday sport-related activities at home. As subjective vitality encompasses both somatic and psychological factors, restrictions resulting from lockdown could drain their overall sense of personal energy. During 2020, a number of studies have been conducted examining the effects of COVID-19 and lockdown on psychological health and well-being. These studies point to a substantial increase in reported psychological problems in the general population (Harper et al., 2020; Taylor et al., 2020) and a significant decrease in well-being (Yang and Ma, 2020). In sport, when comparing athletes’ scores before and during the COVID-19 crisis, research has found an increase in perceived stress (di Fronso et al., 2020) and other ill-being indicators (Lades et al., 2020). The results of the present study confirm the hypothesized negative impact of the COVID-19 lockdown on athletes’ psychological responses with a significant decrease in feelings of personal energy and vitality revealed.

Aligned with prior predictions, a significant decrease in autonomous goal striving emerged when contrasting athletes’ goal motives before the lockdown to what they reported during the lockdown. It seems that the lockdown, an external and uncontrollable “force,” may have had an influence on athletes’ more self-determined reasons for pursuing their sporting goals. Contrary to the hypothesis though, controlled goal motives did not increase from pre-lockdown to lockdown. As they were socially and logistically restricted in their typical activities because of the virus and government restrictions, it was expected that the athletes would view their goals as more regulated by external factors. However, the present findings suggest that among the present sample of athletes, their sport goals as they went from pre-lockdown to during lockdown were not more fueled by controlled reasons. That is, it seems that during this time period, their motives for goal pursuit were less intrinsic and identified (i.e., pursuing the goal because of personal volition, interest, and personally valued implications of achieving the goal) but not more based on extrinsic and identified factors.

Regarding the second hypothesis, a model was tested, which postulated that resilience would predict changes in autonomous and controlled goal motives, which in turn would predict changes in subjective vitality during the time period of interest (i.e., pre-lockdown and during lockdown). In line with the hypothesis, resilience, through its effect on autonomous goal motives, positively and significantly predicted changes in subjective vitality. Athletes with higher resilience showed more pronounced increases in autonomous goal motives over time, and athletes with greater increases in autonomous goal motives exhibited greater increases in subjective vitality over time. Or the other way around, athletes with lower resilience showed greater decreases in autonomous goal motives over time, which in turn corresponded to greater decreases in subjective vitality over time. These results support previous evidence regarding the adaptive impact of resilience in adverse situations and its positive contribution to athletes’ well-being (Gupta and Sudhesh, 2019) and self-regulation (Brown et al., 2015). Further, the present findings are consonant with past studies grounded in the SCM, which have found that autonomous goal motives positively predict well-being (Smith et al., 2011; Healy et al., 2014).

Contrary to the hypothesis, resilience did not predict also changes in controlled goal motives. In spite of the evidence indicating that resilience is linked to diminished ill-being (Sarkar and Fletcher, 2014; Codonhato et al., 2018; Wagstaff et al., 2018), there is less research on the implications of resilience on motivation-related processes that may be operating in any relationship between this personality disposition and well-being or ill-being outcomes. In line with current findings, past research has found that when the predictor was a positive personality variable (e.g., self-oriented perfectionism and resilience), only autonomous motivation was a significant mediator between this personality factor and well-being or ill-being. In previous research and aligned with present results, controlled motivation did not play a significant mediational role (Atienza et al., 2020; León-Guereño et al., 2020). Therefore, results suggest that resilience can act as a promoter of well-being through ensuing increases in autonomous goal motives, without influencing the controlled goal motives over time.

Concerning the interplay between controlled goal motives and well-being, the present results revealed changes in controlled goal motives to negatively predict changes in subjective vitality. That is to say, athletes with greater decreases in controlled goal motives over time exhibited less decrement in reported subjective vitality. Therefore, those athletes who most decreased in their controlled goals during confinement were able to maintain higher levels of subjective vitality. These results are in line with previous evidence that has supported a negative relationship between controlled goal motives and indicators of well-being (Healy et al., 2014; Gaudreau and Braaten, 2016).

The results of the present study correspond to findings of previous sport studies grounded in the SCM. That is, the results are consonant with past findings indicating that autonomous goal motives, and conversely for controlled motivation, will lead to better well-being (e.g., Smith et al., 2007, 2011; Gaudreau and Braaten, 2016) and, specifically, to better subjective vitality (e.g., Healy et al., 2014). Moreover, the present findings contribute to the literature by indicating that autonomous goal motives, as one psychological mechanism, mediate the relationship between resilience and subjective vitality. In regard to study limitations, it should be noted that all the participants were university-level athletes participating in team sports. Further research might explore the relationships of interest in athletes from individual sports and from other age groups. In addition, the results of this study have been obtained in specific circumstances resulting from the COVID-19 pandemic. Thus, caution should be taken in generalizing present findings to other stressful, uncertain, and unusual circumstance. Finally, it should be taken into account that in this research, a relative stability of resilience over time was assumed, that is, the presumed more dispositional nature of this variable (Block and Kremen, 1996; Klohnen et al., 1996; Friborg et al., 2003). For this reason, resilience was only measured once during the study. Other research employing longitudinal designs conducted in other contexts have assessed resilience (as the key dispositional/individual difference factor) only once (e.g., Ayala and Manzano, 2014). In the sport domain, there are limited longitudinal studies examining resilience over time. Existing evidence has not found significant differences when resilience has been measured at different time points during the competitive season (Secades et al., 2016). However, in a recent review, Bryan et al. (2019) found past studies suggesting that resilience can also be more dynamic, which could change and potentially develop through the learning obtained from experiencing adverse situations (McLarnon and Rothstein, 2013). Accordingly, future sport research could consider measuring resilience more than once in order to give light to this matter regarding the assumed stability of athletes’ resilience over time.

Despite these limitations, this study contributes to the knowledge base regarding the benefits of resilience. The findings suggest that through its relationship with autonomous goal motives, resilience is promotive of or at least contributes to the maintenance of athletes’ well-being in spite of adverse situations. Thus, these results confirm the importance of including personal dispositions in the study of self-regulation processes. The present research provided further understanding of the interplay between resilience–goal motives–well-being and as such is relevant to researchers but also informative for sport practitioners who may need to implement interventions with athletes who are struggling during particularly challenging times. During the current COVID-19 pandemic, a wide range of online programs have been available with the aim of maintaining people’s well-being and diminishing any negative impact of the lockdown and uncertainty on psychological health. From an SDT perspective (Deci and Ryan, 1985, 2000), researchers have developed interventions with the objective of promoting well-being during the lockdown. Results suggest that participants who have received the intervention (basic psychological need-satisfying activities during 10 days), self-regulated more autonomously, reported higher well-being, and exhibited lower levels of stress (Behzadnia and FatahModares, 2020). With such findings in mind, these results encourage researchers to specifically develop and implement interventions oriented to promote athletes’ more autonomously regulated goal striving.

## Data Availability Statement

The raw data supporting the conclusions of this article will be made available by the authors, without undue reservation.

## Ethics Statement

The studies involving human participants were reviewed and approved by the University of Valencia. The patients/participants provided their written informed consent to participate in this study.

## Author Contributions

All authors listed have made a substantial, direct and intellectual contribution to the work, and approved it for publication.

## Conflict of Interest

The authors declare that the research was conducted in the absence of any commercial or financial relationships that could be construed as a potential conflict of interest.
